# Effectiveness of a mobile health intervention on uptake of recommended postnatal care services in Nigeria

**DOI:** 10.1371/journal.pone.0238911

**Published:** 2020-09-14

**Authors:** Aanuoluwapo Omobolanle Olajubu, Boluwaji Reuben Fajemilehin, Temitope Oluwafemi Olajubu, Babajide Samuel Afolabi

**Affiliations:** 1 Department of Nursing Science, College of Health Sciences, Obafemi Awolowo University, Ile-Ife, Nigeria; 2 Department of Family Medicine, Obafemi Awolowo University Teaching Hospitals Complex, Ile-Ife, Nigeria; 3 Department of Computer Science, Obafemi Awolowo University, Ile-Ife, Nigeria; Guttmacher Institute, UNITED STATES

## Abstract

Studies have linked the large percentage of maternal and neonatal mortality that occur in postnatal period to low uptake of postnatal care (PNC) services. Mobile health (mHealth) intervention through message reminders has resulted in significant increase in antenatal care utilisation in previous studies. However, its use in PNC services’ uptake has not been adequately investigated in Nigeria. This study aimed to evaluate the effect of a mobile health intervention on PNC attendance among mothers in selected primary healthcare facilities in Osun State, Nigeria. A quasi-experimental research design was utilised. Participants were allocated to Intervention Group and Control Group. One hundred and ninety pregnant mothers were recruited in each group. A mobile health intervention software was developed and used to send educational and reminder messages to mothers in the intervention group from the 35th week of pregnancy to six weeks after delivery. Uptake of PNC services was assessed at birth, 3 days, 10 days and 42 days after delivery. Data were analysed using descriptive statistics, chi-square and logistic regression models. About one-third (30.9%) of respondents in the intervention group had four postnatal care visits while only 3.7% in the control group had four visits (p < 0.001). After controlling for the effect of confounding variables, group membership remained a significant predictor of PNC uptake. (AOR: 10.869, 95% CI: 4.479–26.374). Mobile health intervention significantly improved utilisation of the recommended four postnatal care visits.

## Introduction

Maternal health is considered a major indicator of health as well as the social and economic growth of any country [1, 2]. In developing countries, maternal mortality has been reported as the main challenge of maternal health [3] and usually, a critical measure of advancement in improving maternal health is a reduction in the rate of maternal mortality [4].

In 2015, the global Maternal Mortality Ratio was estimated to be 210 maternal deaths per 100, 000 live births, which is equivalent to 289,000 maternal deaths. Unfortunately, the sub-Saharan Africa region alone accounted for 62% (179, 000) of these global deaths, followed by Southern Asia at 24% (69, 000). At the country level, two countries (India and Nigeria) accounted for one-third of the global maternal death: India at 17% (50, 000) and Nigeria at 14% (40, 000). The report further showed that the MMR in developing regions (230) was 14 times higher than in developed regions [5].

Furthermore, studies have shown that more than half of maternal deaths occurs in the postpartum period [6–8], majority of which occur in sub-Saharan Africa and in particular, Nigeria [9–12]. The major causes of high maternal mortality and morbidity in Nigeria and in several other sub-Saharan African countries comprise poverty, illiteracy, and low use of recognised maternal health care services [13]. Studies have also found that uptake of maternal healthcare services which include postnatal care services is associated with improved maternal and neonatal health outcomes [14–16]. However, despite the various evidence on the importance of postnatal care for reduction of maternal and neonatal mortality, poor uptake of postnatal care services has been reported in various parts of the world [3, 17–19].

Several factors have also been associated with the poor uptake of postnatal care services, the list includes but is not limited to: lack of accessibility, poverty, low education levels, lack of knowledge of pregnancy-related complications, few antenatal care checks, untrained birth attendants, lack of awareness among women with regard to the importance of postnatal care, long waiting time, forgetting appointments, poor quality of services, negative cultural beliefs and dynamics of decision making within the family [17, 18, 20, 21].

Some of the studies in Low and Medium Income Countries (LMIC) show that appointment reminders and educational text messages increased maternal healthcare utilisation among the patients [22–24]. Most of the available studies concentrate mainly on the antenatal period while the postnatal period has received little investigative attention. In Kenya, Fedha [22] assessed the impact of reminders and educational messages sent as Short Message Service (SMS) to mobile phones of pregnant women. Lund *et al*. [24] did a similar study in Zanzibar, while Lau *et al*. [23] utilised SMS to promote healthy antenatal health behaviours among a sample of South African women. In Nigeria, Oyeyemi and Wynn [25] gave mobile phone support to pregnant women and compared their antenatal health facility utilisation with a control group. All these studies reported improved antenatal care attendance.

Although the study by Adanikin *et al*. [20] focused on the role of text messaging in the utilisation of postnatal care services, there were some limitations identified by the author coupled with the fact that the study only assessed utilisation of the sixth week postnatal care visit. The current recommendation is that mothers and babies should have at least four postnatal care contacts with healthcare providers [8].

The availability of phones and use of mobile health (mHealth) have been anticipated as a prospective response to address many of the difficulties that developing countries like Nigeria are confronted with in the healthcare delivery system. These difficulties include: shortage of health personnel, lack of health information, limited training for health care workers, clients forgetting their appointments and difficulty in tracking patients. Worldwide, mHealth projects have been executed using mobile phones for data gathering, record keeping and communication with patients [26]. Also, mHealth technology enables healthcare professionals and institutions to promptly address the critical medical needs of people, especially those in remote locations and those that lack qualified medical personnel and services [27]. The potential value of mHealth deployment in Nigeria is enormous in view of the fact that the country has a mobile phone penetration rate of 84% [28].

However, there is a gap in knowledge and a dearth of information regarding whether mHealth can improve the utilisation of the recommended postnatal care services. Hence, this study determined the effectiveness of mobile health intervention on uptake of the recommended postnatal care services in selected primary healthcare facilities in Osun State, Nigeria.

## Materials and methods

### Design

The study adopted a quasi-experimental design to evaluate the effectiveness of a mobile health intervention on the uptake of postnatal care services among women attending antenatal clinic in the selected primary healthcare facilities in Osun State.

### Study setting

The study was conducted in primary healthcare facilities from six selected Local Government Areas (LGAs) of Osun State, Southwest Nigeria. Osun State is one of the 36 states of the country, it is divided into 30 LGAs for administrative purposes. The map of the study area is as shown in S1 Fig.

The healthcare system in the country is structured along three levels, i.e., primary, secondary and tertiary. The primary healthcare facilities are managed by the LGA administrations. The majority of basic maternal healthcare services takes place at the Primary Healthcare Centres (PHCs). According to the Nigeria Demographic and Health Survey (NDHS), the level of literacy among women in the study area was 91.5% while the baseline postnatal care use was 42% [29].

### Sample size and sampling technique

Sample size was calculated using the sample size formula for comparing two groups [32]. Sample size of 174 was obtained but to allow for 10% attrition rate, a total of 190 was obtained per group. Multistage sampling technique was utilised to select samples for the study. Six LGAs were randomly selected for the study, three of which were assigned to each of the intervention (mobile health intervention) and control (standard care) arms by simple random allocation.

In each of the selected LGAs, all the PHCs that were staffed with at least one registered nurse, provided comprehensive maternal and child healthcare services on a 24-hour basis and had the basic tools for provision of quality postnatal care, were included in the study. In the three LGAs assigned to the intervention arm, a total of nine PHCs met the eligibility criteria and were included in the study, while eight PHCs met the criteria in the control LGAs.

Women who registered for antenatal care (ANC) at the selected PHCs and met the inclusion criteria were recruited for the study and were followed up until 6 weeks after delivery. To be eligible, a woman must be at gestational age of 28–34 weeks, literate, must possess and be able to operate a functional cell phone. The research assistants and study subjects were not blinded because of the nature of the study which required explicit participation.

### Ethics approval and consent to participate

This study was approved by the Health Research Ethics Committee (HREC) of the institute of Public Health, Obafemi Awolowo University, Ile-Ife with assigned number IPHOAU/12/580. Participants were duly informed about the purpose, procedure and proposed duration of the study and they provided verbal and written consent to participate in the study.

### Procedure and data collection

The study was carried out between January and December 2017. The basic demographic data of each enrolled mother was obtained with a structured questionnaire at inclusion. Information about respondents’ obstetric history and other covariates were recorded. There were two language options for participants i.e. English or Yoruba. Mothers in the intervention group also stated their preferred language for text messages. With regards to the distance of their residence, if travelling by foot to the health facility would take more than 15 minutes, it was regarded as far.

In order to ensure that mothers had the baseline information on the WHO-recommended postnatal care visits, all of them received a baseline education on the recommended number of visits including the timing and purpose of each visit. In addition to the standard care and baseline education received by all the mothers, those in the intervention arm received automated text messages (mobile health intervention).

Participants were followed up and a checklist was used to record information about their attendance at the first (day 0–1), second (day 3), third (day 7–14) and fourth (6th week) PNC visits. For the first PNC visit, a woman is said to have utilised the PNC services if she delivered in the health facility and received immediate postnatal care for at least 24 hours or if the birth is at home, she is presented in the facility with her new born for routine check within 24 hours. One of the staff members at each of the selected PHCs was trained and served as research assistant. A trained supervisor was assigned to each LGA who visited all the centres once a week for quality control.

### Mobile health intervention

The mHealth intervention consisted of an automated short messaging service (SMS). A postnatal care assistant software was developed to send text messages to the phone numbers of the mothers. The SMS provided maternal health educational messages and postnatal care attendance reminders. The content of the messages was adapted from MAMA messages [30] with additional input based on literature review and WHO guidelines on postnatal care services [8]. The messages were further reviewed by experts in the field of maternal and child health and also modified based on the feedback from pilot study. All the messages were translated to Yoruba, the predominant local language, by back-translation method. Hence, they were available to the participants in either English or Yoruba depending on their preference. All the participants could speak and read either of the two languages.

The topics covered include: healthy prenatal lifestyle practices, medical signs for seeking clinical care, the recommended number and timing of postnatal care visits among others. A breakdown of the types, frequencies, timing and samples of the educational and reminder messages are shown in Table 1. The intervention began at 35 weeks gestational age until six weeks post-partum. PNC attendance reminders were also sent a day prior to each of the four expected visits. Based on the gestational age of each woman, the software created an algorithm for the schedule of the messages. When a participant in either of the study groups delivered, she sent a prearranged code via SMS, free of charge, indicating that she had given birth. The software thereafter began to send postnatal care messages to those in the intervention arm. When this code had not been received from a participant by their EGA of 42 weeks and efforts to contact them proved abortive, they were deemed to have been lost to follow up.

**Table 1:** Schedule and samples of educational and reminder messages.

Category	Aspects covered	Frequencies and Timing	Examples
Educational	• PNC	***Before birth*:**	*‘After the delivery of your baby you are expected to have 4 postnatal contacts*: *the first is to stay in the healthcare facility where you deliver for 24–48 hour*.*’ (week 35)*
	• Healthy self-care	Week 35 until birth: 2 messages per week	
	• Danger signs and indications for seeking care		
	• Breastfeeding		
	• Cord-care		
	• Immunisation		
		***After birth*:**	*‘Some bleeding after birth is normal*. *But if you soak more than 2–3 pads in 20 minutes or the bleeding gets heavier*, *go to the clinic*.*’ (Day 2)*
		Week 1: 3 messages (days 2,3,5)	
		Weeks 2–6: 2 messages per week (i.e., between day 1 to 41)	*‘Are you irritable and weepy*? *This is quite common among new mothers*. *Get some rest*, *make sure you eat well and report in the clinic for proper assessment’*. *(Day 12)*
Reminders		Postnatal days 1, 2, 9 and 41	*‘Congrats*, *your baby's here*! *If you delivered at home*, *please come immediately with the baby to the health facility for the first postnatal contact*.*’ (Day 1)*

### Outcomes

The aim of this study was to evaluate the effect of a mobile health intervention on the rate of utilisation of postnatal care services. We used the WHO recommendation of four postnatal care visits at day 1, i.e., within 24 hours of birth, day 3 (second visit), between days 7–14 (third visit) and week 6 (fourth visit). The main outcome measured was the proportion of women who attended all the four postnatal care visits (yes or no). The following outcomes were also assessed: rate of attendance at each of the four visits and the total number of PNC visits utilised.

### Statistical analysis

Stata software (version 14.0) was used for the analyses. Descriptive statistics were used in summarising the socio-demographic data and postnatal care attendance of the respondents while Pearson Chi square was used to compare the baseline characteristics in the two groups.

Appropriate regression models were used to assess the impact of mobile health intervention on the outcome measures. Logistic regression models were applied for binary outcome variables, i.e., attendance of all four postnatal care visits (‘yes’ or ‘no’) by mothers in each of the study groups, and attendance at each of the four recommended PNC visits. Negative binomial regression model was used for count outcomes, i.e., the total number of PNC visits attended by the mothers.

In order to control the baseline differences, the variables that were statistically different between the two study groups were retained in the final models. For these multivariate analyses, some of the socio-demographic variables were dichotomised by a merger of some sub-categories which had low frequency counts.

To account for the clustering nature of the data, generalised estimating equations were used in all logistic regression analyses [31]. The principle of complete case analyses was used for the final analyses thus excluding those with incomplete follow-up data. Attrition analyses was conducted using chi-square tests for categorical variables and independent t tests for continuous variables. The significance level for all the analyses was set at p ≤ 0.05.

## Results

**Fig 1:**
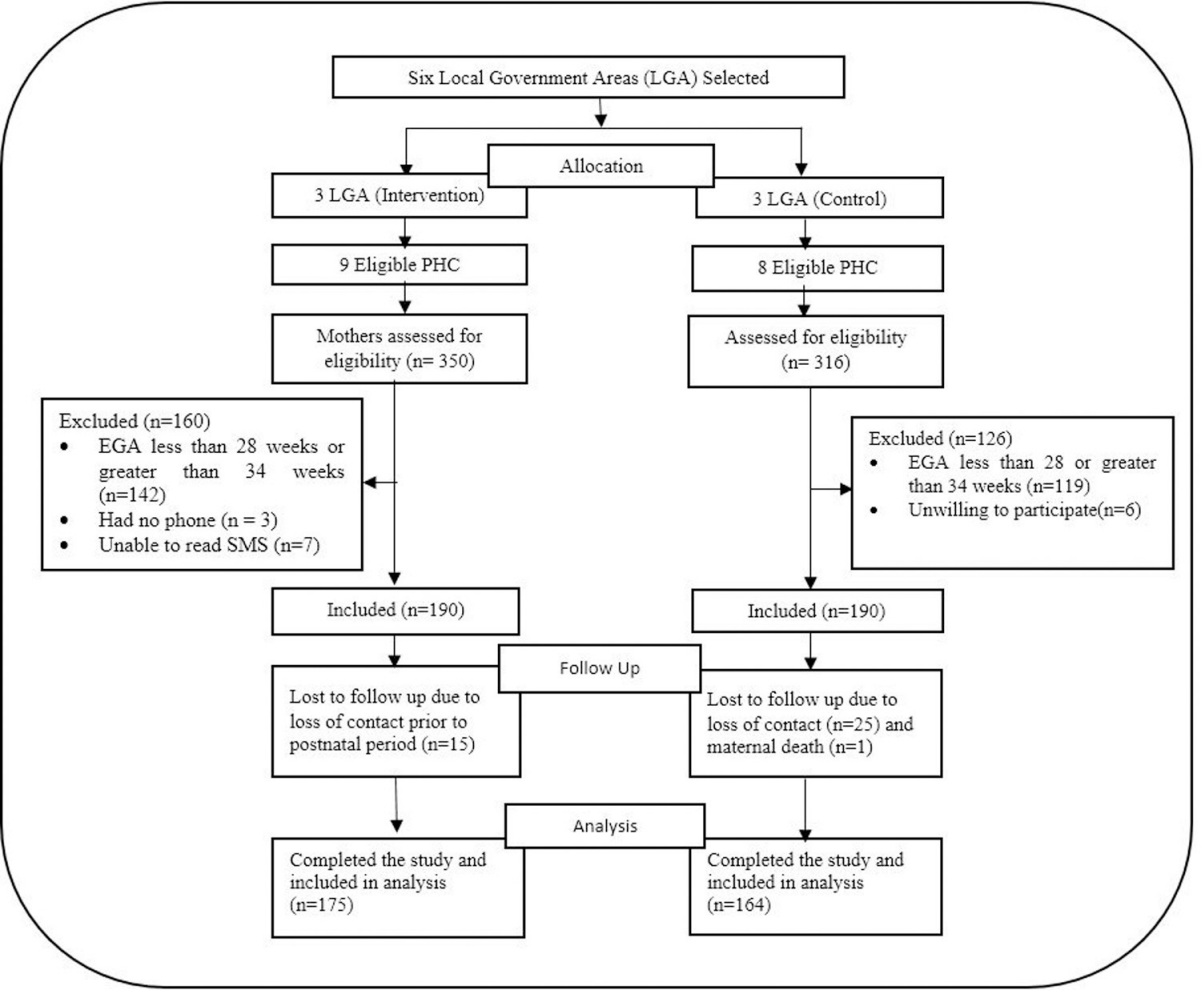
Flowchart of study participants.

Fig 1 presents the flowchart of the participants. A total of 380 women were recruited, 190 in each of the study groups. Among these, 15 were lost to follow up in the intervention group and 26 in the control group, giving a completion rate of 92.1% (n = 175) and 86.3% (n = 164) respectively. The attrition analyses showed that the respondents lost to follow up did not differ significantly from those who completed the study. Those with complete follow up data were included in the final data analyses.

### Baseline characteristics

Table 2 highlights and compares the baseline socio-demographic variables of the two groups. Majority of the respondents in both the intervention (62.6%) and control (77.4%) groups were within the age range of 18–30 years. The two groups were similar in most of the baseline variables except with respect to their age (*p* = 0.001), educational level (*p* = 0.001), partner’s educational level (*p* = 0.01) and partner’s occupation (*p* = 0.004). This was factored into consideration in subsequent analyses.

**Table 2:** Respondent’s baseline characteristics.

Variables	Intervention (n = 190)	Control (n = 190)	P value
**Age (years)**			
**Mean age**	29.54 ± 4.99	27.75 ± 4.73	
**18–30**	119 (62.6)	147 (77.4)	
**31 and above**	71 (37.4)	43 (22.6)	0.002
**Marital Status**			
**No Partner**	5 (2.6)	7 (3.7)	0.385
**Has Partner**	185 (97.4)	183 (96.3)	
**Tribe**			
**Yoruba**	168 (88.4)	177 (93.2)	0.088
**Hausa**	11 (5.8)	10 (5.3)	
**Igbo**	11 (5.8)	3 (1.6)	
**Educational Attainment**			
**Primary**	21 (11.0)	37 (19.5)	0.001
**Secondary**	144 (75.8)	107 (56.3)	
**Tertiary**	25 (13.2)	46 (24.2)	
**Partner’s Educational Attainment**			
**Primary**	13 (6.8)	21 (11.0)	0.010
**Secondary**	128 (67.4)	98 (51.6)	
**Tertiary**	49 (25.8)	71 (37.4)	
**Occupation**			
**Civil servant**	11 (5.8)	24 (12.6)	0.133
**Trader**	110 (57.9)	98 (51.6)	
**Artisan**	55 (28.9)	53 (27.9)	
**Unemployed**	14 (7.4)	15 (7.9)	
**Partner’s Occupation**			
**Civil servant**	21 (11.1)	38 (20.0)	0.004
**Trader**	57 (30.0)	68 (35.8)	
**Artisan**	92 (48.4)	77 (40.5)	
**Unemployed**	20 (10.5)	7 (3.7)	
**Income**			
**Below 18,000**	133 (70.0)	139 (73.2)	0.285
**18,000 & above**	57 (30.0)	51 (26.8)	
**Gravidity**			
**2–3**	138 (72.6)	144 75.8)	0.279
**4 and above**	52 (27.4)	46 (24.2)	
**Parity**			
**0–2**	178 (93.7)	180 94.7)	0.413
**3 and above**	12 (6.3)	10 (5.3)	
**House distance to the facility**			
**Near**	170 (89.5)	141(74.2)	0.100
**Far**	20 (10.5)	49 (25.8)	

### Impact of mHealth intervention on PNC uptake

In addition to the study group membership (i.e., intervention status) as the variable of interest, all the variables in Table 2 were included in the first logistic model as potential confounders during model selection. Thereafter, non-significant confounders were removed with the use of backward elimination which resulted in study group membership as the only significant predictor. However, the variables with baseline differences between the two groups were retained in the final logistic regression model in addition to the study group membership.

After adjusting for the potential confounders and clustering effect, the odds for utilising four postnatal care visits was about 11 times higher for women who received the mobile health intervention compared to those in the control group. (30.9% vs. 3.7%, AOR: 10.869, 95% CI: 4.479–26.374). (Table 3)

**Table 3:** Association between intervention status and uptake of four PNC visits.

	Four PNC Visits				
	Yes	No	AOR*	95% CI	*P* value
	n (%)	n (%)			
**Study group**					
Intervention	54 (30.9)	121 (69.1)	10.869	4.479–26.374	<0.001
Control	6 (3.7)	158 (96.3)	1		
**Age group**					
31 years & above	86 (79.6)	22 (20.4)	1.001	0.531–1.884	0.997
18–30 years	193 (83.5)	38 (16.5)	1		
**Highest Education**					
Secondary and above	235 (81.0)	55 (19.0)	1.553	0.504–4.783	0.443
Primary	44 (89.8)	5 (10.2)	1		
**Partner’s Highest education**					
Secondary and above	252 (81.8)	56 (18.2)	0.934	0.263–3.316	0.916
Primary	27 (87.1)	5 (12.9)	1		
**Partner’s Occupational Status**					
Employed	264 (83.8)	51 (16.2)	0.438	0.170–1.129	0.087
Unemployed	15 (62.5)	9 (37.5)	1		

*Adjusted for clustering effect and potential confounders.

We further used the attendance at each of the four postnatal care visits as outcome variables and applied same analytic procedure as above. The summary of the result is as presented in Table 4.

**Table 4:** Association between mHealth intervention and utilisation of each PNC visit.

	Utilisation		AOR*	95% Confidence Interval	P value
	Intervention	Control	(Intervention vs. control)		
	n (%)	n (%)			
First PNC	134 (76.6)	65 (39.6)	5.122	3.142–8.347	< 0.001
Second PNC	78 (44.6)	13 (7.9)	9.261	4.794–17.888	< 0.001
Third PNC	67 (38.3)	13 (7.9)	6.215	3.226–11.974	< 0.001
Fourth PNC	149 (85.1)	108 (65.9)	2.749	1.595–4.738	< 0.001

*Adjusted for within-cluster effect and baseline group differences in: age, education, partner’s education and occupation.

It showed that for each of the recommended visits, the odds of utilisation were significantly higher among the mothers in the intervention arm. The odds ratio was highest with regard to the second PNC visit which was utilised by 44.6% of those in the intervention group compared to 7.9% in the control arm (AOR: 9.261, 95% CI: 4.794–17.888) and lowest for the fourth visit (85.1% vs. 65.9%, AOR: 2.749, 95% CI: 1.595–4.738).

In the same vein, a negative binomial analysis of the total count of visits by each mother revealed that, for those in the intervention group, the incident rate of attendance was almost double that of their control counterparts. (Incident Rate Ratio: 1.966, 95% CI: 1.486–2.602).

## Discussion

The objective of this study was to evaluate the effectiveness of a mobile health intervention on the recommended four postnatal care attendance among mothers in selected primary healthcare facilities in Osun State, Nigeria.

The introduction of mHealth intervention in the form of appointment reminders and educational text messages had a significantly positive impact on the spectrum of outcome measures of PNC utilisation among the respondents. The proportion of women who utilised the recommended four visits in the intervention group were significantly higher than those who did in the control group. The odds for utilising the four PNC visits was about 11 times higher for women who received the mobile health intervention compared to their control counterparts. For each of the PNC visits, the same trend of higher odds of attendance was demonstrated by mothers in intervention group. This is in consonance with report from the study of Adanikin *et al*. [20] which found that patients who received SMS reminders were significantly more likely to attend their postnatal clinic appointment.

Many other studies in low and medium income countries (LMIC) have shown the same trend of improvement in uptake of different aspects of maternal healthcare services after introduction of mHealth tools especially in the form of educational messages and appointment reminders [22–25, 32]. In their respective randomised controlled trials, Lund *et al*. [24] in Tanzania and Fedha [22] in Kenya, reported significant increase in antenatal care attendance among women who received relevant text messages. Da Costa *et al*. [32] in a study conducted in Brazil, reported a significant reduction in non-attendance rates in three of the four clinics studied after the introduction of SMS appointment reminders. Similarly, a South African study showed statistical evidence that SMS promoted positive healthy behaviours among pregnant women in terms of facility utilisation [23].

The positive impact of the text messages deployed in this present study could have been mediated by a couple of factors. Apart from serving as appointment reminders which has been shown to trigger an increase in positive response by patients and clients in different clinical settings [20, 22–25, 32], the educational component potentially helped to impress on mothers the importance and benefits of routine PNC utilisation. There have been reports demonstrating positive association between mothers’ knowledge about PNC care and services and their uptake of such services [10, 33, 34].

It is noteworthy that, to the best of our knowledge, this is the first study in the country which assessed the impact of mHealth intervention on utilisation of the WHO-recommended four PNC visits [8], the study by Adanikin et al. [20] and other PNC-related studies focused on the traditional 6th week visit. However, the importance of the four-visit recommended schedule cannot be overemphasised [35, 36]. It has been shown that majority of preventable maternal morbidity and mortality occur in the first few hours to days into the post-partum period [21]. Hence, routine check-ups in the earlier part of the postnatal period is crucial for the purpose of receiving life-saving preventive, diagnostic and interventional care. This, without gainsaying, has the potential of reducing maternal mortality especially in countries like Nigeria where the burden remains unacceptably high. This can quicken the journey to the achievement of sustainable development goal (SDG) three [37].

As this study has shown, mHealth intervention is one of the measures that can promote the utilisation of these recommended visits. Although Da Costa *et al*. [32] raised the issue of ethical implications and limitations about sending SMS reminders to patients, they argued that a message sent to a patient’s cell phone can be read by others and cause embarrassment to the patient. However, this might not be of significance in the case in postnatal care services which is associated with a joyous context of care after childbirth. Studies [38, 39] have also indicated several advantages of SMS over voice call which include but not limited to: availability of SMS on approximately 98% of mobile phones, cheaper in cost, minimal technical expertise is required for its use and its adaptability to multiple mHealth purposes. Also, messages can be accessed at user’s convenience and can be delivered to phones that are turned off or have flat batteries.

Therefore, the findings from this study strongly support the submission [40] that more attention should be accorded the deployment of SMS messages as a potential measure in enhancing healthcare delivery.

### Implications for future research

The usefulness of mHealth intervention in improving postnatal care attendance requires its acceptance and adequate ICT skills among healthcare workers (HCW). Hence, the attitude, capacity, and practice of healthcare workers regarding mHealth for effective postnatal care need to be explored. This will help to document the current state of mHealth use among healthcare workers. Furthermore, the potential impact of appropriate training in improving HCW’s knowledge and capacity to deploy mHealth intervention needs to be examined. The impact of mHealth intervention on specific maternal health outcomes, i.e., in terms of morbidity and mortality, is worthy of investigation in order to provide possible further evidence in support of its adoption by policy makers.

### Study limitations

One of the limitations encountered in the course of the study was the erratic power supply which occasionally delayed the delivery to some respondents whose phones were off due to flat battery. Also, the lack of blinding could have potentially caused some response bias especially by the respondents in the intervention group. The study was geographically restricted to six Local Government Areas in a state in Nigeria. Caution needs to be taken regarding inferences to mothers outside the study settings.

## Conclusion

This study concluded that mHealth intervention in the form of education and reminder SMS has the potential of improving postnatal care uptake. Therefore, mHealth intervention is recommended for deployment in maternal healthcare services especially postnatal care.

## Supporting information

S1 Fig Map of the study locations. (PDF)
