# Supplementary material for: Effectiveness of a mobile health intervention on uptake of recommended postnatal care services in Nigeria
Source: PLoS One. 2020 Sep 14;15(9):e0238911. doi: 10.1371/journal.pone.0238911 (PMC7489550; doi:10.1371/journal.pone.0238911)
Supplement: S1 Fig — (PDF) [file pone.0238911.s001.pdf]

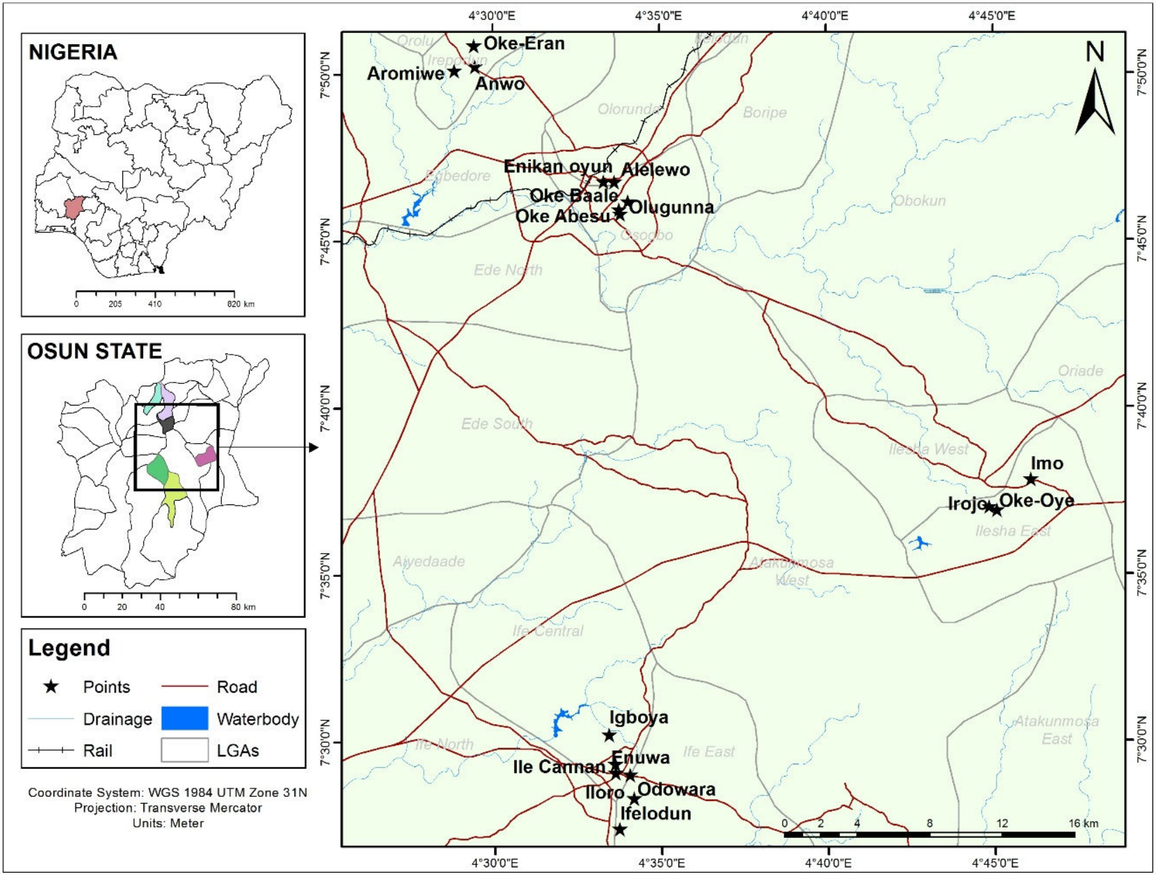

NIGERIA
0
205
410
820 km
OSUN STATE
0
20
40
80 km
Legend
Points
Road
Drainage
Waterbody
Rail
LGAs
Coordinate System: WGS 1984 UTM Zone 31N
Projection: Transverse Mercator
Units: Meter
4°30'0"E
4°35'0"E
4°40'0"E
4°45'0"E
7°50'0"N
7°45'0"N
7°40'0"N
7°35'0"N
7°30'0"N
N
Oke-Eran
Aromiwe
Anwo
Olorunda
Boripe
Enikan oyun
Alelewo
Egbedore
Oke Baale
Olugunna
Oke Abesu
Obokun
Osogbo
Ede North
Oriade
Ede South
Ilesha West
Imo
Irojo
Oke-Oye
Ilesha East
Aiyedaade
Atakunmosa West
Ife Central
Igboya
Atakunmosa East
Ife North
Ife East
Ile Cannan
Enuwa
Iloro
Odowara
Ifelodun
0
2
4
8
12
16 km
